# Cohort profile: a community-based prospective cohort study of Alzheimer’s disease and related dementias in the Democratic Republic of Congo

**DOI:** 10.1002/bsa3.70085

**Published:** 2026-06-06

**Authors:** Jean Ikanga, Caterina Obenauf, Saranya Sundaram Patel, Megan Schwinne, Guy Gikelekele, Emmanuel Epenge, Japhet Magolu Potshi, Topina Tomadia, Julie Kazadi, Immaculee Kavugho, Giovanni Luamba Santolini, Aristote Nkosi, Francine Manyonga Sabowa, Brenda Matungu Musewu, Elipsis Mawisa, Joseph Phuati Tsangu, Francis Muena Kondo Beya, Samuel Mampunza, Lelo Mananga, Justine Bukabau, Charlotte E. Teunissen, Thomas Karikari, Alden L. Gross, Julio C. Rojas, Alvaro Alonso

**Affiliations:** 1 Department of Rehabilitation Medicine, Emory University School of Medicine, Atlanta, Georgia, USA; 2 Department of Psychiatry, School of Medicine, University of Kinshasa and Catholic University of Congo, Kinshasa, Democratic Republic of Congo; 3 Department of Psychology and Neuroscience, University of Tennessee, Knoxville, Tennessee, USA; 4 One Rehab, Dallas, Texas, USA; 5 School of Medicine, Department of Biomedical Informatics, Emory University, Atlanta, Georgia, USA; 6 Department of Neurology, University of Kinshasa, Kinshasa, Democratic Republic of Congo; 7 Memory Clinic of Kinshasa, Kinshasa, Democratic Republic of Congo; 8 Department of psychology, University of Kinshasa, Kinshasa, Democratic Republic of Congo; 9 Department of Anesthesiology and Reanimation, University of Kinshasa, Kinshasa, Democratic Republic of Congo; 10 Department of Internal Medicine, University of Kinshasa, Kinshasa, Democratic Republic of Congo; 11 Amsterdam University Medical Centers, Neurochemistry Laboratory, Department of Clinical Chemistry, Amsterdam Neuroscience, Neurodegeneration, Amsterdam University Medical Centers, Vrije Universitiet, Amsterdam, the Netherlands; 12 Department of Psychiatry, School of Medicine, University of Pittsburg, Pittsburg, Pennsylvania, USA; 13 Department of Epidemiology, Johns Hopkins Bloomberg School of Public Health, Baltimore, Maryland, USA; 14 Memory and Aging Center, Weill Institute for Neurosciences, Department of Neurology, University of San Francisco, San Francisco, California, USA; 15 Department of Epidemiology, Rollins School of Public Health, Emory University, Atlanta, Georgia, USA

**Keywords:** Alzheimer’s disease, biomarkers, cohort study, dementia, Democratic Republic of Congo

## Abstract

**INTRODUCTION:**

The Étude du Vieillissement Cognitif et de Démence en République Démocratique du Congo (EVCD-RDC) launched in 2024 to investigate socioenvironmental and biological determinants of dementia in Kinshasa.

**METHODS:**

The study recruited 1000 adults aged ≥65 years across four diagnostic groups – cognitively unimpaired (CU), mild cognitive impairment (MCI), Alzheimer’s disease (AD), and other dementias (OD) – through community and clinical settings. Participants complete neuropsychological, neurological, social, and environmental assessments with biennial follow-up.

**RESULTS:**

To date, 506 individuals have enrolled (mean age 73.4; 59.3% women), including 70 CU, 136 MCI, 120 AD, and 180 with OD. Dementia cases show higher rates of hypertension, comorbidities, neuropsychiatric symptoms, smoking, and alcohol use, along with lower body weight, education, and resilience, and greater exposure to pollution, conflict, trauma, and poverty.

**DISCUSSION:**

EVCD-RDC is the first large-scale dementia cohort in French-speaking sub-Saharan Africa and will provide critical longitudinal insights into modifiable risks, infectious diseases, and multi-omics in the region.

## 1 | INTRODUCTION

Cognitive aging and dementia are emerging global public health concerns due to aging populations worldwide. Recent estimates indicate that more than 50 million people are affected by dementia globally; this figure is projected to nearly triple by 2050, disproportionately impacting low- and middle-income countries (LMICs) due to population growth and aging.^1^ The increasing burden of dementia and cognitive aging issues not only overwhelms healthcare systems and social services, but it also places a financial and emotional burden on individuals, families, and communities, with global costs beyond $1 trillion annually.^2^ Identifying modifiable risk factors and improving early and accurate diagnosis are therefore critical priorities for reducing the future burden of dementia.

As the global population ages, the rising incidence of dementia necessitates a thorough investigation of its evolving epidemiology and treatment approaches. Large-scale epidemiologic studies suggest that addressing modifiable risk factors, such as education and cardiovascular risk factors, could substantially reduce dementia incidence globally.^3^ However, most evidence informing these estimates comes from high-income, Western countries, limiting generalizability to settings where risk profiles, healthcare access, and cultural contexts differ markedly. In many LMICs, poverty, limited access to preventive healthcare, and a higher burden of untreated vascular risk factors may fundamentally alter dementia risk trajectories. In parallel, empirical advances in dementia biomarkers have improved diagnostic accuracy.^3^ Similarly, biomarker testing offers a biologically grounded approach to identifying neurodegenerative disease and distinguishing dementia subtypes, but biomarker research has been conducted almost exclusively in high-income countries. As a result, little is known about how biological markers of neurodegeneration interact with socioeconomic and cultural factors in LMICs or whether biomarker thresholds observed elsewhere are applicable in these settings.

Longitudinal cohort studies have proven particularly valuable for addressing these gaps, as they allow for monitoring of the prevalence, incidence, and progression of cognitive impairment over time.^3, 4^ In high-income countries, well-established programs such as the Framingham Heart Study in the United States^5^ and the Whitehall II Study in the United Kingdom^6^ have provided critical insights into the risk factors and progression of cognitive aging. These cohorts examine how modifiable cardiovascular, metabolic, and social risk factors, including hypertension, hyperlipidemia, smoking, obesity, diabetes, physical inactivity, and occupational socioeconomic status, contribute to cognitive aging and dementia over time. Cohort studies in LMICs, including the 10/66 Dementia Research Group studies in Asia and Latin America^7^ and the Brazilian Longitudinal Study of Aging (ELSI-Brazil),^8^ have similarly aimed to characterize dementia prevalence and incidence while evaluating the influence of education, socioeconomic adversity, health behaviors, and access to healthcare on cognitive aging and dementia, and findings from these cohorts are beginning to reveal unique epidemiologic patterns driven by differing socioeconomic and lifestyle factors relative to high-income countries. In sub-Saharan Africa (SSA), non-French-speaking countries such as Nigeria and South Africa have initiated cohort studies to explore dementia and aging.^9,10^ However, research in French-speaking African countries remains limited, with few prospective cohort studies conducted in these contexts.^11^ Prevalence and risk factors of dementia have been examined in the French-speaking Republic of Congo,^12^ Central African Republic,^13^ and Benin.^14^ In the Democratic Republic of the Congo (DRC) specifically, there have also been cross-sectional studies,^15^ but no longitudinal cohort studies, reflecting complex challenges such as linguistic and cultural barriers to research.^16^

To aid in addressing this gap, we have established the Étude du Vieillissement Cognitif et de Démence en République Démocratique du Congo (EVCD-RDC; Study of Cognitive Aging and Dementia in the DRC), which is recruiting and prospectively following a representative sample of older adults in the French-speaking DRC to investigate the clinical course, biomarkers, and neuropsychiatric symptoms (NPSs) of cognitive aging and dementia within this understudied population. Consistent with prior longitudinal dementia cohorts, the overarching objectives of EVCD-RDC are to examine cognitive trajectories over time and to evaluate how socioeconomic conditions, vascular and metabolic health, NPSs, and biological markers contribute to dementia risk. The objective of this cohort profile paper is distinct from these longitudinal aims; it aims to describe the EVCD-RDC cohort and methodology. Although this study is longitudinal in nature, most of the objectives (i.e., objectives 1 to 4) are intended to be achieved in future phases. This paper presents only the initial descriptive results from an ongoing longitudinal study. Specifically, we describe the origins, study design, and key enrollment characteristics of this unique cohort and provide a cross-sectional overview of baseline findings. This cohort profile therefore lays the groundwork for future longitudinal studies that aim to inform prevention and treatment programs not only in the DRC but also in similar contexts across French-speaking Africa.

## 2 | SUBJECTS/MATERIALS AND METHODS

### 2.1 | Cohort description

#### 2.1.1 | Cohort setting, objectives, and design

The EVCD-RDC is a prospective, longitudinal cohort study based in Kinshasa, the capital of the DRC.

The key objectives of this cohort study are to (1) estimate prevalence, incidence, and risk factors for cognitive aging and dementia; (2) track longitudinal changes in functional, behavioral, and cognitive symptoms, as well as neurodegenerative biomarkers, over time; (3) validate neurodegenerative biomarkers and establish cutoffs supporting early dementia diagnosis, disease monitoring, and treatment response; (4) develop and refine diagnostic criteria, predictive models, and prognostic tools in this new population; and (5) assess the needs of individuals with dementia and the burden on caregivers and family members to inform strategies for caregiving and provide evidence-based recommendations for healthcare policy, practice, and guidelines.

### 2.2 | Overall study design

The study design includes four phases: (1) Recruitment and Enrollment: Participants were recruited through churches, senior associations, military camps, hospitals, and private clinics. Community leaders shared a written announcement describing eligibility criteria, study importance, and relevance to older adults. Interested volunteers were screened by the research team. Recruitment was most successful in churches and senior associations, where community leaders played a key supportive role. In contrast, recruitment in military camps, hospitals, and private clinics was less successful due to extensive administrative requirements for obtaining approval to recruit participants. In churches and senior associations, community leaders were briefed on the importance of the study for the country, the medical community, their own communities, and individual participants. In many communities, we also held 1- to 2-h educational sessions on healthy aging, age-related changes, dementia, and caregiving for individuals with dementia. This approach had a strong impact on community engagement and resulted in a high number of volunteers enrolling in our cohort study.

**FIGURE 1:**
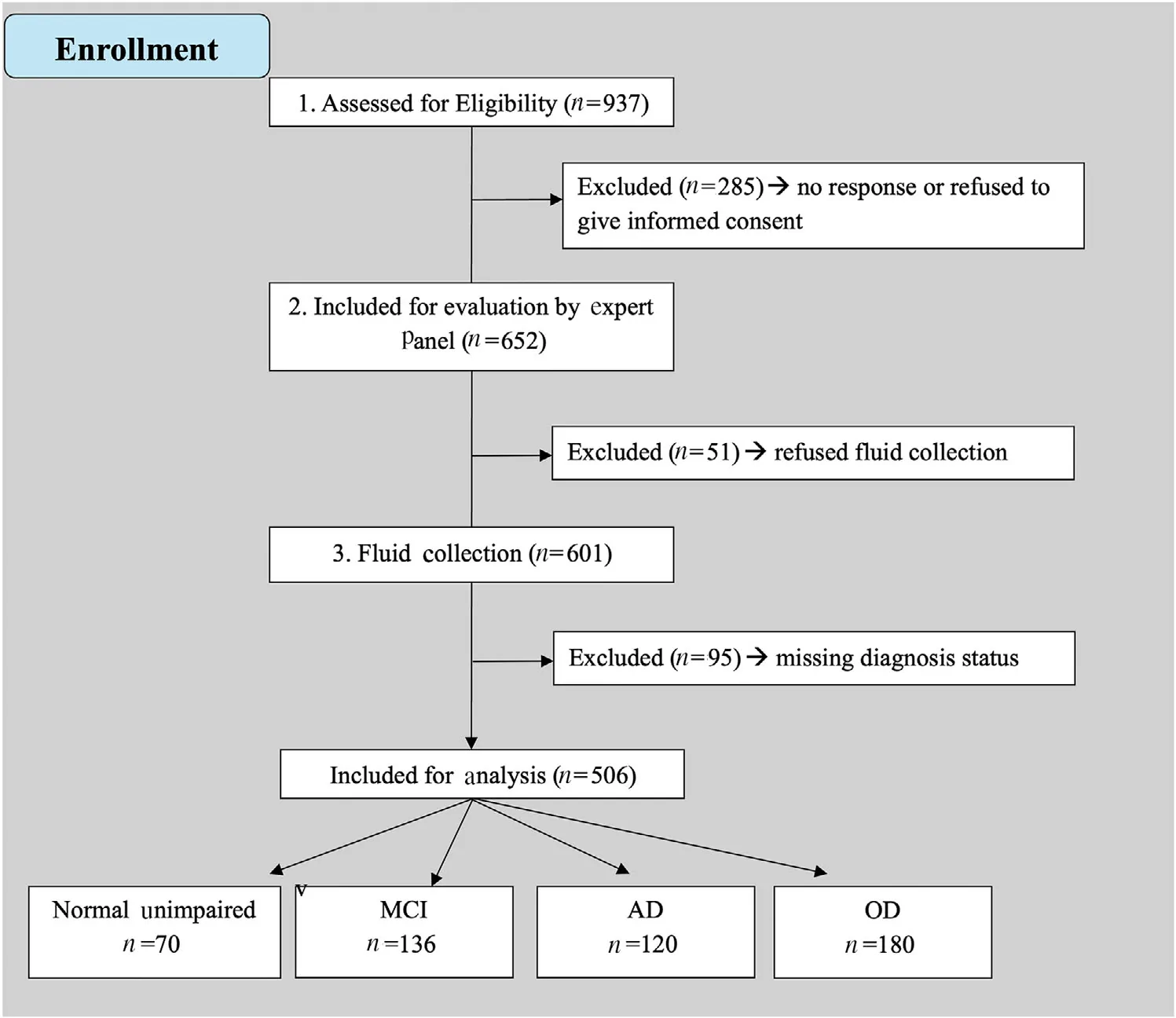
Clinical adjudication process for classifying participants as cognitively unimpaired (CU), mild cognitive impairment (MCI), suspected Alzheimer’s disease (AD), or other dementia (OD). The process involved two steps: (1) determining presence of cognitive deficits and (2) evaluating possible etiology based on neuropsychological performance, functional status (IDEA), medical history, and neurological/psychiatric evaluations. Classifications were adjudicated by a multidisciplinary team (neuropsychology, neurology, psychiatry, and internal medicine).

(2) Screening and Evaluation: Screening tests, questionnaires, and neurocognitive assessments were conducted at recruitment sites. Screening, neurocognitive assessments, and questionnaires were conducted by clinical psychologists with training in cognitive assessment, psychometrics, and the evaluation of older adults with and without dementia, using tablets and REDCap. Screening tests included the Montreal Cognitive Assessment (MoCA),^17^ the Clinical Dementia Rating (CDR®) Dementia Staging Instrument, ^18^ and the Identification and Intervention for Dementia in Elderly Africans (IDEA) study’s measure of Instrumental Activities of Daily Living (IADLs) in elderly Africans. ^19^ After participants recruited in Phase 1 and administered screening tests in Phase 2, participants were excluded from the current study if they expressed concerns about the screening tests being too long, not liking the screening tests, and wishes to withdraw (see Step 1 in Figure 1).

Physical, clinical, and neurological evaluations were performed by psychiatry and neurology residents who were also trained in the medical assessment of older adults. All staff involved in data collection were members of the local community and were familiar with the language, culture, and stigma surrounding dementia. Although no formal certification process was in place, we ensured the development of relevant skills and expertise by requiring each evaluator to assess five volunteers under supervision and to receive positive feedback from their supervisor.

(3) Sample Collection and Neurological Evaluation: Biological samples (cerebrospinal fluid [CSF], blood, urine, saliva, feces) and neurological exams were performed at Centre Medical de Kinshasa (CMK) to avoid misconceptions about specimen collection outside clinical settings.

(4) Cohort Follow-Up: Follow-ups – including cognitive testing, neurological exams, and specimen collection – occur every 2 years. The initial phase (recruitment to collection) spanned August 2024 to September 2025, with the first follow-up planned for August 2026, pending funding. The figure summarizes study procedures (Figure 1).

#### 2.2.1 | Cohort eligibility

Eligibility was determined during recruitment and screening visits (Figure 1). To be eligible for inclusion in this open cohort, individuals must (1) be Congolese citizens residing in the DRC; (2) be 65 years or older; (3) provide a valid home address, phone number, and the contact information of a caregiver or informant; (4) agree to participate longitudinally (including baseline and follow-ups); (5) have the ability to provide informed consent, either independently or via a caregiver; (6) be fluent in Lingala or French; and (7) have no prior neurodevelopmental, psychiatric, or neurological disorders affecting the central nervous system. Individuals with significant vision or auditory impairments were excluded.

### 2.3 | Study measurements

Measures included the Uniform Data Set version 3 (UDS-3), screening tools, the African Neuropsychology Battery-Short Version (ANB-SV), intelligence tests, and other questionnaires. These tests were derived from previous dementia cohort studies^20,21^ to harmonize results across cohorts and align with international studies. Additional tools addressed context-specific aging factors in SSA – resilience, poverty, illiteracy, air pollution, and cardiovascular health. Resilience and poverty indices were computed from questionnaire totals. All instruments were culturally adapted, translated into French and Lingala, and pre-piloted before implementation (Table S1). Neurological evaluation included cranial nerves, gait and balance, coordination, reflexes, muscle strength and tone, and sensation. Neurological evaluation was crucial to rule out other neurological disorders causing cognitive deficits (e.g., stroke, tumors) as well as other types of dementia (e.g., Parkinson’s disease, Lewy body dementia). The follow-up assessments are outlined in Table S1.

### 2.4 | Clinical adjudication process

Participants were classified as cognitively unimpaired (CU), mild cognitive impairment (MCI), suspected Alzheimer’s disease (AD), or other dementia (OD). A multidisciplinary team (neuropsychologist JI, neurologist EE/PJ, psychiatrist GG, internist TT) conducted adjudication in two steps: (1) determining cognitive status (CU vs not CU) and (2) identifying etiology based on impairment pattern, medical history, neurological findings, and lifestyle. Following Manly et al.,^22^ participants with no cognitive domain impairment and normal functioning (identification and IDEA cognitive screen ≥ 30) were classified as CU. Deficits in one domain with preserved function (IDEA ≥ 30) indicated MCI. Impairment in at least domains (> 1.5 SD below mean) with functional loss (IDEA < 30) indicated dementia. Progressive, memory-predominant decline defined suspected AD, whereas stepwise decline, fluctuating performance, medical causes, or personality change indicated OD (Figure 1).

To determine the predicted score of the participant, we applied a linear regression model using scores from screening, functional, cognitive, and intelligence tests. This model examined the relationship between predictors and cognitive outcomes. Outcome variables were screening, cognitive, intelligence, and functional tests; predictors were age, gender, and years of education. Our regression equation was as follows:

(1) Y%=%β0+β1%×%1+β2%×%2+β3%×%3

where Y represents screening test scores, cognitive test scores, functional test scores, or intelligence test scores; X1 = education; X2 = age; and X3 = gender.

The linear regression equation was Y=β0+β1(education)+β2(age)+β3(gender). The coefficients β0,β1,β2, and β3 were obtained based on our previous study.^15^ Model fit was evaluated using $R^{2}$, with interpretation guided by Congolese clinical context. Analyses considered predictor strength, significance, and the model’s predictive ability for cognitive decline or dementia.

Participant impairment was quantified with *z*-scores using CU test data from our prior study^15^:

(2) z%=%(X-μ)/σ

where X is the participant’s predicted score based on the linear regression equation, μ represents the population mean, and σ denotes the population standard deviation.

#### 2.4.1 | Collection of biological fluid

Various biological fluids were collected from participants following international standards at CMK by trained laboratory technicians. CSF was collected from 200 participants (50 in each category). We explained to all volunteers the importance of CSF in the diagnosis of dementia. They were informed that positron emission tomography (PET) scans and CSF are considered the gold standard, rather than blood tests, for a valid and sensitive diagnosis of AD. Since PET scans were not available, we requested volunteers for CSF collection. We maintained records in each group to ensure that we had 50 participants, in line with the requirements of the central limit theorem. CSF was collected in sterile polypropylene tubes, non-hemolyzed blood via venipuncture. Blood (for plasma, serum, PAXgene RNA, and DNA), urine, saliva, and feces were collected from all participants. The blood collection process included (1) a 10-cc dry tube without anticoagulant for serum; (2) three EDTA tubes with anticoagulant: one 10-cc tube for plasma and two 4-cc tubes for DNA; and (3) an additional 2.5 mL of blood, which was drawn into a PAXgene Blood RNA tube containing an intracellular RNA stabilizer.

Saliva was collected with Sarstedt Salivette tubes (using a cotton roll saturated for ~2 min) and urine in Sarstedt bottles after fluid restriction; fecal samples were home-collected.

##### Centrifugation and aliquoting

Blood (serum/plasma), CSF, and urine underwent centrifugation and aliquoting in sterile tubes. Blood collected without anticoagulants was left at room temperature for at least 30 min before processing.

The centrifugation steps were as follows:

- Blood, CSF, and urine were centrifuged at 4000 rpm for 10 min.
- Processed specimens were placed on a rack and pipetted into 0.5-mL (500 μL) aliquots to obtain serum and plasma.
- Urine was transferred to sterile 6-mL conical tubes before centrifugation.
- PAXgene Blood RNA, saliva, and feces were not centrifuged.

#### 2.4.2 | Sample storage

CSF, plasma, serum, and urine aliquots were stored at −20°C then −80°C for biomarker stability. Saliva was stored immediately; feces and PAXgene RNA was held at −20°C for 24 h then −80°C for long-term storage.

#### 2.4.3 | Pre-analytic and biobanking

Procedures ensured (1) sample quality, (2) reduced error and variability, (3) minimized false results, (4) workflow efficiency, and (5) regulatory compliance. Technicians labeled samples by ID, fluid type, classification, and date and logged tube type, centrifugation, and storage details, including time between collection, centrifugation, and storage at −20°C/−80°C. A −80°C freezer was acquired to enhance sample integrity and management.

#### 2.4.4 | Estimating kidney function

Because kidney function affects circulating biomarkers (e.g., amyloid beta [A*β*], tau), the estimated glomerular filtration rate (eGFR) was included as a covariate.^23^ To assess kidney function in our sample, we calculated eGFR using serum creatinine levels. Bukabau and colleagues^24,25^ found that commonly used eGFR equations (Chronic Kidney Disease Epidemiology Collaboration, the European Kidney Function Consortium, and the revised Lund-Malmo Revised), even those adjusted for race, were not applicable to SSA populations. Based on their previous findings, we calculated the eGFR using the Modification of Diet in Renal Disease (MDRD) formula, as our serum creatinine measurements were not calibrated. After adjusting for demographic factors such as age and gender, the eGFR was calculated using the following formula:

$$eGFR\;(MDRD)=175\times {\left(\text{Serum}\;\text{creatinine}\right)}^{-1.154}\times (\text{Age}{)}^{-0.203}\times (0.742\;if\;female).$$

This formula is commonly used to monitor kidney function and detect chronic kidney disease.^24,25^

#### 2.4.5 | Air pollution

Previous epidemiological studies ^26^ showed a significant association between air pollution and dementia, with research suggesting that long-term exposure to poor air quality increases the risk of cognitive decline and dementia. In LMICs, individuals have prolonged exposure to various types of air pollution, including indoor, outdoor, occupational, community, and natural sources. ^27^ Therefore, we collected data on select pollutants to assess both cross-sectional and longitudinal links between air pollution, cognitive deficits, and dementia. Air pollution, linked to neuroinflammation, oxidative stress, and endothelial dysfunction, was measured using an ATMO Tube PRO device capturing PM_1_, PM_2·5_, PM_10_, volatile organic compounds, barometric pressure, temperature, humidity, and altitude.^28–30^ Data collectors were trained by a team expert on how to use the ATMO Tube PRO device. Each collector wore the device for 1 h inside the participant’s residence, measuring air pollutants every 15 min. A total of four readings were recorded. These readings were summed and averaged to estimate the air pollution level for each participant.

### 2.5 | Data management

Data were collected and managed in REDCap^31,32^ by trained staff entering sociodemographic, clinical, cognitive, and biological variables. Automated quality rules flagged errors, duplicates, and missing values, which were reviewed and corrected by the data team. Following final review, de-identified data were exported to SPSS version 29 for analysis. Variables were recoded and scored per guidelines. Missing data (<5%) were handled by pairwise deletion after descriptive evaluation.

### 2.6 | Cohort profile analysis

Descriptive statistics (mean ± SD, frequency percentage) were computed by neurological status. Multinomial logistic regressions adjusted for age, sex, and education assessed associations with neurological status. Likelihood-ratio tests (LRTs) compared full versus reduced models. ANOVA and logistic models (with LRT *p* values) examined continuous and binary outcomes, respectively. Tukey’s post hoc tests were conducted for all pairwise comparisons, with the neurological status of CU as the reference group. Statistical significance was defined as p < 0.05. All analyses were performed using R (version 5). ^33^

## 3 | RESULTS

### 3.1 | Characteristics of cohorts

Table 1 presents sociodemographic characteristics by neurological status. To date, the cohort includes 506 participants (mean age = 73.4, SD = 5.9); 59.3% are women, including 70 CU (14%), 136 MCI (27%), 120 AD (24%), and 180 OD (36%). Mean education is 9.7 years (SD = 4.8); CU and MCI have significantly more education than suspected dementia (AD/OD). Most participants are Bantu (98%), with smaller Nilotic/Sudanic representation and no Pygmy participants. Suspected dementia was associated with higher divorce rates, whereas CU/MCI were more often married. All participants lived with either a spouse or a relative. Most lived independently, but suspected dementia required more help with complex daily activities than CU/MCI. Nearly all participants resided in non-assisted living homes (Table 1).

### 3.2 | Medical history of cohort

Table 2 summarizes the medical history of the cohort. Average CU weight was 78.3 kg; suspected dementia weighed less (AD = 68.6 kg; OD = 64.0 kg). Mean body mass index (BMI) was 25.9 kg/m^2^, lower in suspected dementia. Hypertension was common (64.9% aware, 30.8% treated, 31.7% controlled). Neurological abnormalities occurred in 21% overall, highest in OD (31%). Common conditions included ear, nose, and throat (ENT) disorders (38%), musculoskeletal (21%), vascular (17%), and upper GI (12%) disorders. Creatinine was stable across groups (mean 0.90, median 0.85 mg/dL). Mean eGFR (69.7 to 74.4 mL/min/1.73 m^2^) was below normal (>90).

### 3.3 | Neuropsychiatric profile of cohort

Table 2 also presents NPSs and questionnaire results. Nearly half of the participants (44%) had experienced war, and 59% reported traumatic events within the past 10 years. Only 12% were tobacco smokers, and 21% consumed alcohol about once a month. Sleep-related issues were reported by 31% of the sample, including sleep apnea, sleep disorders, or insomnia (Table 2). Suspected dementia had a more adverse social determinants of health (SDOH) profile than CU/MCI. Although the overall cohort had a high resiliency score (mean = 19.9), the suspected dementia group scored lower (18.0) than CU (21.3). Poverty scores were higher in suspected dementia (6.8) versus CU (4.8) and MCI (5.8).

**FIGURE 2:**
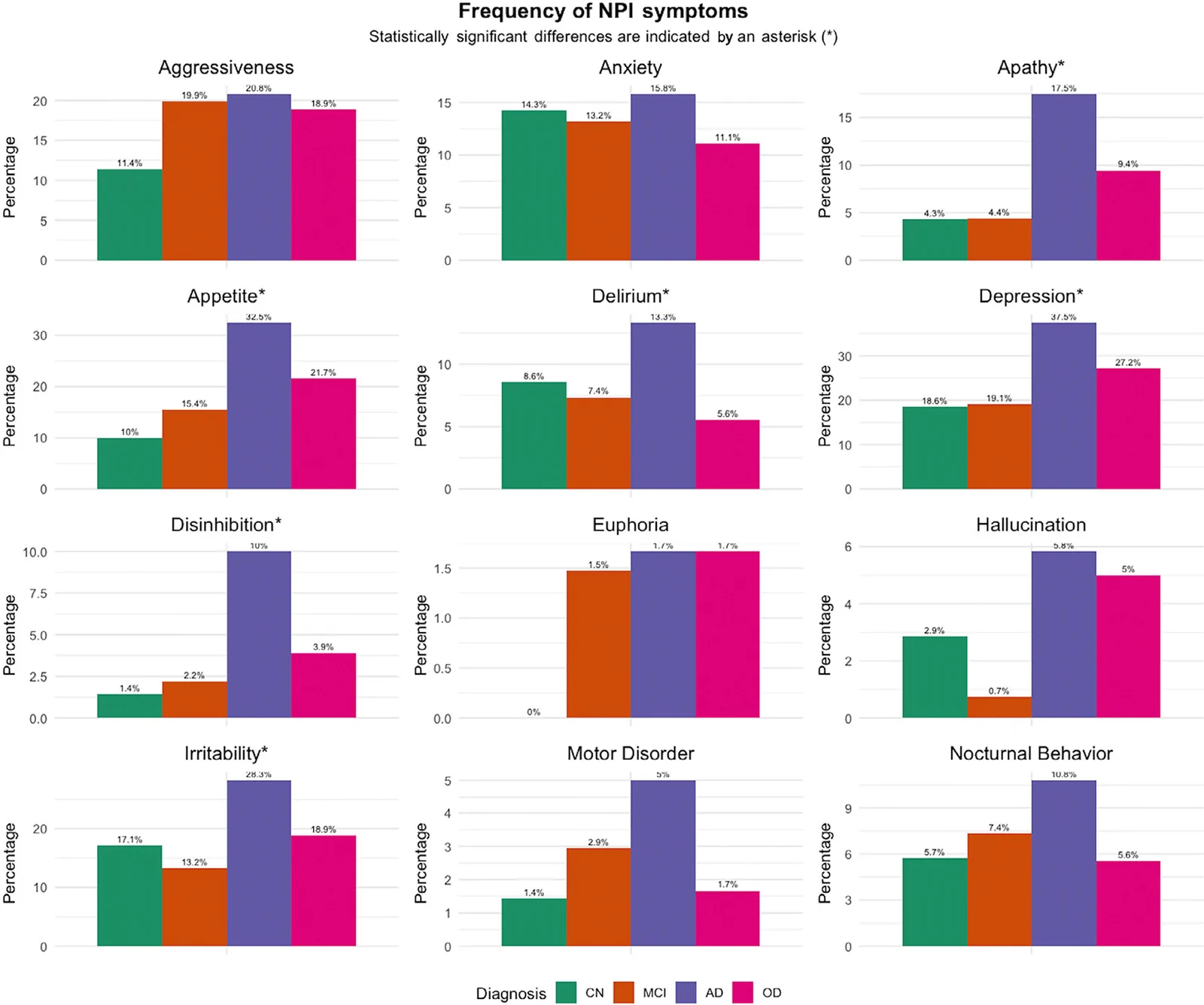
Frequency of neuropsychiatric symptoms as measured by the Neuropsychiatric Inventory (NPI) among EVCD-RDC participants, stratified by diagnostic group.

The most common NPSs were depression (26%), loss of appetite (21%), irritability (19%), and aggressiveness (19%), with higher prevalence in AD than other groups. Participants with suspected dementia exhibited a higher frequency of NPSs, with those diagnosed with suspected AD showing more symptoms than individuals with other types of dementia. Statistically significant differences in NPS frequency were observed between groups, particularly in apathy, appetite changes, delirium, depression, disinhibition, and irritability (Figure 2).

### 3.4 | Cognitive profile of cohort

Table S2 summarizes the psychometric properties (internal consistency) of screening and cognitive tests. Most tests showed good to excellent Cronbach’s *α*. The African Visuospatial Memory Test and African Proverb Test showed acceptable reliability. The internal consistency of the African Emotion Test was questionable (0.441).

Table 3 presents the screening and neuropsychological test results. Most of the screening and cognitive tests, including measures of intelligence, showed statistically significant differences across groups, with CU participants scoring highest on most tests, followed by those with MCI, OD, and AD. Participants with suspected AD performed more poorly than those with OD, particularly in learning curves and retention. Mean scores differed across all assessments (*p* = 0.001), with CU > MCI/AD/OD.

### 3.5 | Air pollution

**FIGURE 3:**
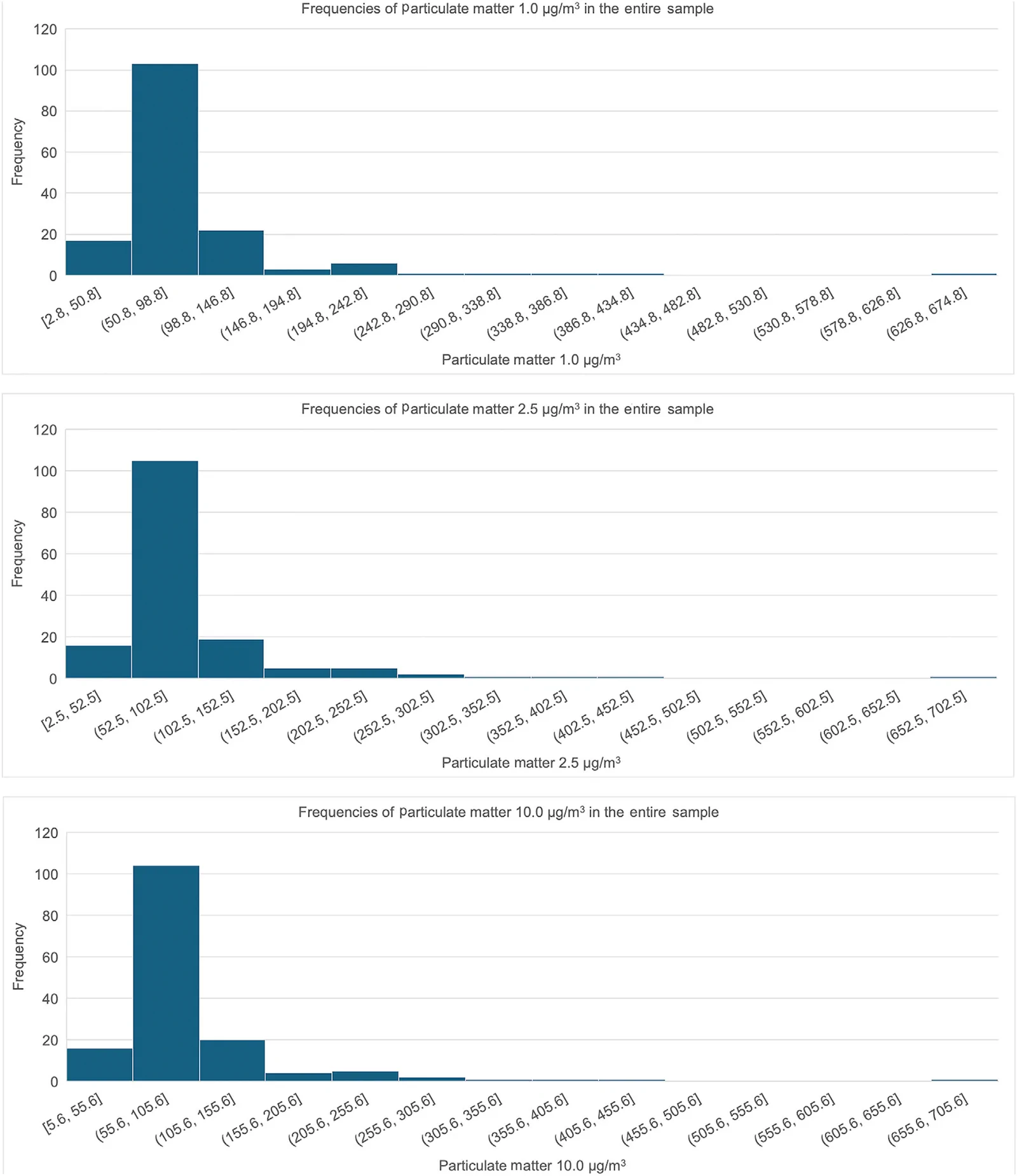
Average concentrations of major air pollutants (e.g., PM_2·5_, PM_10_, NO_2_, and SO_2_) are depicted across monitoring sites in Kinshasa, Democratic Republic of Congo.

Figure 3 depicts air pollution levels in Kinshasa. Concentrations of PM_1_, PM_2·5_, and PM_10_ ranged from 2.8 to 670 μg/m^3^ (mean = 90.73 μg/m^3^, SD = 73.17), 2.5 to 691.3 μg/m^3^ (mean = 102.32 μg/m^3^, SD = 104.21), and 5.6 to 692.3 μg/m^3^ (mean = 98.41 μg/m^3^, SD = 77.36), respectively. No statistically significant differences were observed among diagnostic groups for any of the air pollutants. Barometric pressure (mean 980.30 mmHg, SD 11.58), coefficient of variation (CV) (mean 0.13, SD 0.06), temperature (mean 25.59°C, SD 2.56), humidity (mean 58.10%, SD 6.67), and altitude (mean 278.51 m, SD 101.70) were stable across groups.

## 4 | DISCUSSION

This longitudinal community-based cohort enrolled 506 individuals from 21 out of 24 quarters of Kinshasa, the capital of the DRC. The cohort is geographically, sociodemographically, and linguistically diverse, including participants classified as CU, MCI, AD, or OD, although there may be group-classification issues based on clinical characteristics, as some features associated with ODs can also be present in AD. Ethnically, the cohort mirrors Kinshasa’s demographics of ~80% Bantu with smaller Sudanic, Nilotic, and Pygmy groups.^34^ Additionally, individuals in this cohort with suspected dementia had lower educational attainment compared to those CU and with MCI. As emphasized by the 2024 Lancet Commission, low education is a known risk factor for dementia.^3^

Demographically, this cohort is older (mean age: 73.4 years) than those in some previous studies, such as the Health and Aging in Africa: Longitudinal Study of an INDEPTH Community (HAALSI), where the average age was around 61 years, with a focus on individuals aged 40 and older.^35^ As seen in many aging and dementia cohorts worldwide (e.g., HAALSI, where 53.6% of 5059 participants were women), women are more represented in this cohort, likely due to their longer life expectancy, greater willingness to participate in research, and higher healthcare-seeking behavior. ^35^ Additionally, recruitment through churches may have contributed to this gender imbalance, as women tend to attend church more frequently than men.^36^

Consistent with our previous study,^15^ suspected dementia was also associated with the status of being divorced. Divorce among older adults in Africa can lead to social stigma or shame, financial dependence on family members, limited access to resources, strained family relationships and inheritance issues, as well as challenges related to cultural expectations around aging and family roles. ^37^ These factors may increase vulnerability among divorced older adults and contribute to mental health challenges that could elevate the risk of developing dementia.^37^ The living arrangements observed in this cohort highlight the social importance of multigenerational households in Africa, which provide a supportive environment for older adults. Younger family members often care for the elderly, fostering intergenerational bonds and offering assistance during illness.^38^ In contrast, individuals with dementia in Western countries often reside in private homes with caregivers, assisted living facilities, nursing homes, or other community-based settings.^39^ In Congo, most individuals with dementia live in multigenerational homes without formal or professional assistance. Those with suspected dementia required more help with complex daily activities compared to the CU and MCI groups.

Medically, while SDOH affect adults globally, our cohort faced fewer SDOH-related challenges compared to some Western populations.^40^ Poverty plays a significant role in shaping health outcomes, cognitive aging, and dementia risk in this cohort. Future research should consider poverty as an additional factor in models predicting cognitive functioning. However, it is important to clearly define poverty and specify the criteria used to identify individuals considered poor in LMICs. Unlike many global cohorts, where few participants have experienced war (e.g., in the United States, only 6.1% of adults are veterans),^41^ nearly half of our participants (43.5%) reported wartime experience. According to Sixsmith et al.,^42^ such experiences may foster adaptability, independence, and resilience – traits reflected in participants’ resilience scores. However, Burnes et al.^43^ highlighted the detrimental effects of stress and trauma, which can accelerate neurological damage and increase the risk of cognitive decline and dementia. Future studies should consider the impact of SDOH on screening, diagnosis, and treatment in this sample. Poverty and limited education have a significant influence on performance on many cognitive tests and scores, as these assessments often require a certain level of literacy.^44,45^ Therefore, adapting and developing applicable screening tools is essential to avoid conflating poverty and low education with cognitive deficits or dementia. Poverty and low education also restrict access to healthcare facilities and screening services.^46^ In addition, low health literacy in this sample may have affected awareness of available screening tests, while financial constraints contributed to delayed screening. Finally, the diagnosis of cognitive deficits or dementia has been associated with cultural beliefs and stigma, which may delay or complicate treatment and care. ^47^

Cognitively, validated tools utilized in the current study showed high reliability (Cronbach’s *α*), except for the African Visuospatial Memory, Proverb, and Emotion Tests, which were weaker. Specifically, our findings indicate that most of the cognitive tests demonstrate good reliability (i.e., the test items measure the same underline cognitive function), homogeneity of items (i.e., items are related to each other and measure a single cognitive function), and consistency of results. In contrast, the African Visuospatial Memory Test, the African Proverb Test, and the Emotion Recognition Test show weaker associations with their target cognitive functions. Ikanga and colleagues^48^ similarly reported poor psychometric properties for the African Proverb Test. Finally, the weaker reliability of the Emotion Recognition Test may reflect the fact that many Congolese individuals are not accustomed to labeling emotions in a highly differentiated manner.

Regarding NPS, prior studies categorized them into three clusters: affective symptoms (depression, anxiety, apathy, sleep, and appetite disturbances), agitation symptoms (disinhibition, agitation, irritability, motor disturbances, and euphoria), and psychotic symptoms (hallucinations and delusions).^49^ In our cohort, affective symptoms were the most prevalent, followed by agitation, with few or no psychotic symptoms. As in previous studies, NPSs were more frequent among individuals with suspected dementia than in those who were CU or showed MCI.^50^

Regarding OD risk factors, the prevalence of tobacco smoking in this cohort aligns more closely with rates in the United States^51^ and SSA, where adult smoking prevalence averages 12.7%.^52^ A meta-analysis found that smoking rates among adults aged 65 and older in SSA ranged from 1.8% (Zambia) to 25.8% (Sierra Leone), with men smoking more than women.^53^ Sleep disorders, another dementia risk factor as chronic sleep disruption contributes to neurodegenerative processes and cognitive decline, were also prevalent.^54^ While approximately 40% of older adults globally report poor sleep, individuals with AD experience more sleep disturbances due to comorbidities.^55^ In our cohort, sleep disorder prevalence ranged from 27% to 59%, consistent with global estimates of 14% to 59%.^55^ A meta-analysis suggested that sleep disturbances may predict future dementia risk: insomnia was linked to increased AD incidence, while sleep-disordered breathing was associated with all-cause dementia, AD, and vascular dementia.^56^ Low body weight and BMI in this cohort may reflect poor appetite and confounding factors such as smoking. Weight loss is commonly observed in dementia patients, though the underlying mechanisms remain unclear.^56,57^ Consistent with the existing literature, our findings show a high prevalence of vascular conditions, particularly hypertension, which plays a significant role in cognitive decline and dementia.^58^ Additionally, the cohort’s eGFR indicated mildly reduced kidney function, which may affect biomarker clearance and plasma concentration levels. Therefore, eGFR should be considered in statistical analyses involving biomarkers.

Regarding environmental risk factors, this study confirms recent research identifying Kinshasa – a megacity with over 17 million residents, projected to reach 35 million by 2040 – as the “most polluted major city in the world.”^59^ The city’s air quality exceeds the World Health Organization (WHO) guideline of 5 μg/m^3^ for PM_2.5_, contributing to numerous health issues and an estimated 32,000 deaths annually in the DRC.^60^ The primary contributors to poor air quality include widespread biomass burning, elevated emissions from household charcoal stoves, and persistent environmental contaminants such as arsenic, lead, dichlorodiphenyltrichloroethane derivatives, and polychlorinated biphenyls.^59^ In 2019, McFarlane and colleagues^60^ deployed a five-node PurpleAir monitoring network and reported a calibrated annual average PM_2.5_ concentration of 43.5 μg/m^3^ in Kinshasa. Our findings align with earlier studies showing that PM_2.5_ levels peak during the dry season - June, July, and August - when most of our data were collected. ^61^ Therefore, air pollution should be recognized as a significant and modifiable risk factor for dementia in the DRC.

In sum, the 2024 Lancet Commission outlined 12 modifiable risk factors for dementia across the life course, grouped into lifestyle factors (physical inactivity, smoking, excessive alcohol consumption), health conditions (obesity, hypertension, diabetes, depression), environmental factors (air pollution, traumatic brain injury), and cognitive/social factors (low education, social isolation, hearing impairment).^3,58,62^ Our cohort reported several of these risk factors, particularly health condition (hypertension and depression), cognitive/social factors (low education and hearing impairment), and environmental (air pollution). Most of these modifiable dementia risk factors are late-life ones that can be prevented.

The limitations of this study include its being restricted to Kinshasa, financial constraints delaying −80°C storage and neuroimaging, a few tests with modest reliability, and anticipated attrition affecting power and retention. The study population is community-based, but including a convenience sample, which may pose risks of unrepresentativeness, variability in data quality and consistency, potential biases in participant selection, challenges in controlling for confounding variables, and issues related to stigma and fear of dementia diagnosis. Additionally, the limited sample size of male participants compared to female participants may affect the interpretation and reporting of the results, especially within the cultural context of the DRC. Cultural barriers contributing to low male participation and help-seeking behaviors include cultural expectations regarding masculinity, which includes emphasizing strength and self-reliance, and avoiding being perceived as vulnerable. Further, men are often expected to seek traditional healing practices and may fear participating in research to avoid being accused of witchcraft.^63^ To improve male participation and better examine gender dynamics and inclusion, we recommend recruitment efforts in military camps, universities (where many professors are male), local community or village gatherings, through community leaders, cultural events, sports associations (such as football), and outreach programs. Initiatives should emphasize raising awareness that dementia is a neurological disorder rather than a spiritual disease, and that it affects both men and women. Finally, most of the objectives of this study were not achieved within the scope of this paper; we were unable to fully address our five main objectives, as the majority are longitudinal in nature (i.e., objectives 1 to 4). Moreover, we have not yet analyzed portions of our available data that correspond to some of these objectives (specifically parts of objectives 1, 3, and 4).

The strengths of this study include (1) the feasibility of large-scale dementia research in a LMIC despite cultural barriers to biospecimen collection; (2) generation of rare multi-omics samples from SSA populations; (3) identification of modifiable/non-modifiable dementia factors to guide prevention; (4) interdisciplinary collaboration building national capacity and public awareness that challenges beliefs linking dementia to witchcraft (through this project, we established a national team of experts in cognitive aging and dementia; the team includes clinical psychologists, neuropsychologists, nurses, laboratory technicians, psychiatrists, neurologists, and anesthesiologists, and during the recruitment process, we clarified that dementia is a biological condition rather than a spiritual disorder); (5) prospective design enabling causal inference and rigorous analytic control (the cohort we have built will allow us to collect longitudinal data. This will enable us to track outcomes and examine the effects of various factors on dementia, thereby helping us establish strong evidence for causal relationships between these factors and dementia).

Future plans of our cohort study include the possibility to conduct a clinical trial based on the US Study to Protect Brain Health Through Lifestyle Intervention to Reduce Risk (POINTER) or the Finnish Geriatric Intervention Study to Prevent Cognitive Impairment and Disability (FINGER) models, incorporating both therapeutic and lifestyle interventions to reduce and prevent dementia in SSA. FINGER^64^ and POINTER, which is a similar study in the United States,^65^ are multidomain interventions that combine pharmacological and nonpharmacological approaches, including structured lifestyle programs with regular moderate- to high-intensity physical exercise, adherence to the Mediterranean-DASH (Dietary Approaches to Stop Hypertension) Intervention for Neurodegenerative Delay (MIND) diet, cognitive training and social engagement, and cardiovascular health monitoring. These interventions have demonstrated significant improvements in global cognition and reductions in dementia risk among older adults.^64,65^ We also intend to expand the cohort of EVCD-RDC beyond Kinshasa to examine incidence, mortality rates, and survival outcomes of dementia in the DRC. Improving the psychometric properties of the three less reliable cognitive tests is a priority. Given the high prevalence of wartime trauma, infectious diseases, vascular conditions, sleep disorders, and air pollution in our sample, we aim to investigate their contributions to dementia risk. Finally, we plan to develop cognitive and plasma biomarker profiles across the spectrum of cognitive aging, dementia stages, and subtypes in the DRC. In future publications, we plan to harmonize our locally validated cognitive tests (ANB) with the harmonized cognitive assessment protocol using common items from both instruments. This harmonization will support meta-analyses and data sharing, improve diagnostic accuracy, enhance research collaboration, and inform global health policies.

## Supplementary Material

Supplementary Table 1

Supplementary Table 2

SUPPORTING INFORMATION

Additional supporting information can be found online in the Supporting Information section at the end of this article.

## Figures and Tables

**TABLE 1:** Characteristics of the cohort stratified by neurological status.

Variables, mean (SD)	Overall (*n* = 506)	CU (*n* = 70; 14%)	MCI (*n* = 136; 27%)	AD (*n* = 120; 24%)	OD (*n* = 180; 36%)	*p* value

**Demographics and social determinants of health**						
**Age**	73.4 (5.9)	71.6 (5.1)	72.6 (5.4)	75.2 (6.7)	73.7 (5.7)	0.13
**Female** *	300 (59%)	33 (47%)	80 (59%)	64 (53%)	123 (68%)	0.11
**Linguistic group** *						0.97
Lingala	82 (16%)	12 (17%)	22 (16%)	17 (14%)	31 (17%)	
Kikongo	165 (33%)	28 (40%)	41 (30%)	39 (33%)	57 (32%)	
Swahili	30 (5.9%)	4 (5.7%)	11 (8.1%)	7 (5.8%)	8 (4.4%)	
Tshiluba	74 (15%)	11 (16%)	21 (15%)	15 (13%)	27 (15%)	
Other	155 (31%)	15 (21%)	41 (30%)	42 (35%)	57 (32%)	
**Ethnic group** *						0.63
Bantu	497 (98%)	70 (100%)	132 (97%)	118 (98%)	177 (98%)	
Nilotique	3 (0.59%)	0 (0%)	1 (0.74%)	1 (0.83%)	1 (0.56%)	
Soudanique	4 (0.79%)	0 (0%)	2 (1.5%)	0 (0%)	2 (1.1%)	
Other	2 (0.40%)	0 (0%)	1 (0.74%)	1 (0.83%)	0 (0%)	
**Education, years**	9.7 (4.8)	12.1 (4.1)	10.7 (4.3)	8.8 (5.0)^†^	8.7 (5.0)^†^	0.013
**Marital status** *						0.074
Married	216 (43%)	39 (56%)	70 (52%)	44 (37%)	63 (35%)	
Widowed	247 (49%)	24 (34%)	55 (40%)	61 (51%)	107 (59%)	
Divorced	16 (3.2%)	4 (5.7%)	4 (2.9%)	5 (4.2%)	3 (1.7%)	
Separated	8 (1.6%)	0 (0%)	2 (1.5%)	3 (2.5%)	3 (1.7%)	
Single	14 (2.8%)	2 (2.9%)	4 (2.9%)	4 (3.3%)	4 (2.2%)	
Living with a partner	2 (0.40%)	0 (0%)	0 (0%)	2 (1.7%)	0 (0%)	
Unknown	3 (0.59%)	1 (1.4%)	1 (0.74%)	1 (0.83%)	0 (0%)	
**Living arrangement** *						0.0006
Lives alone	13 (2.6%)	1 (1.4%)	4 (2.9%)	5 (4.2%)	3 (1.7%)	
Lives with spouse/partner	196 (39%)	38 (54%)	60 (44%)	33 (28%)	65 (36%)	
Lives with relative/friend	194 (38%)	24 (34%)	49 (36%)	46 (38%)	75 (42%)	
Lives with caregiver	1 (0.20%)	0 (0%)	0 (0%)	1 (0.83%)	0 (0%)	
Lives with a group	98 (19%)	7 (10%)	22 (16%)	32 (27%)	37 (21%)	
Lives in a group home	2 (0.40%)	0 (0%)	0 (0%)	2 (1.7%)	0 (0%)	
Unknown	2 (0.40%)	0 (0%)	0 (0%)	1 (0.83%)	0 (0%)	
**Level of independence** *						< 0.0001
Able to live independently	311 (62%)	59 (84%)	100 (74%)	56 (47%)	96 (53%)	
Needs help with complex activities	150 (30%)	10 (14%)	30 (22%)	42 (35%)^†^	68 (38%)^†^	
Needs help with basic activities	34 (6.7%)	0 (0%)	3 (2.2%)	18 (15%)	13 (7.2%)	
Completely dependent	9 (1.8%)	1 (1.4%)	2 (1.5%)	3 (2.5%)	3 (1.7%)	
Unknown	2 (0.40%)	0 (0%)	1 (0.74%)	1 (0.83%)	0 (0%)	
**Type of residence** *						0.64
Private residence	501 (99%)	70 (100%)	134 (99%)	118 (98%)	179 (99%)	
Assisted living facility	2 (0.40%)	0 (0%)	0 (0%)	1 (0.83%)	1 (0.56%)	
Unknown	3 (0.59%)	0 (0%)	2 (1.5%)	1 (0.83%)	0 (0%)	

Abbreviations: AD, Alzheimer’s disease; CU, cognitively unimpaired; MCI, mild cognitive impairment;OD, other dementia.

* Results presented as n (%).

† Indicates significant pairwise association between the neurological status category and cognitively normal (the reference group).

**TABLE 2:** Medical and neuropsychiatric profiles of cohort, stratified by neurological status.

Variables, mean (SD)	Overall (*n* = 506)	CUD (*n* = 70; 14%)	MCI (*n* = 136; 27%)	AD (*n* = 120; 24%)	OD (*n* = 180; 36%)	*p*-value

**Medical**						
**Physical evaluation**						
Height, cm	162.6 (8.6)	165.4 (7.4)	165.0 (8.5)	161.6 (9.3)	160.4 (8.0)	0.11
Weight, kg	69.6 (18.5)	78.3 (25.4)	73.2 (19.5)	68.6 (17.9)	64.0 (12.8)^†^	0.025
BMI, kg/m^2^	25.9 (5.1)	26.3 (4.5)	27.2 (5.2)	24.9 (4.6)	26.2 (6.9)	0.10
Systolic BP, mmHg	141.6 (21.9)	147.9 (21.7)	137.0 (21.1)^†^	138.4 (22.8)^†^	144.3 (21.2)	0.009
Diastolic BP, mmHg	85.5 (12.9)	86.3 (11.6)	84.8 (13.0)	84.4 (13.0)	86.2 (13.5)	0.69
Stroke history*	11 (2.2%)	0 (0%)	2 (1.5%)	3 (2.5%)	6 (3.3%)	0.41
Heart rate, bpm	72.4 (10.7)	72.1 (9.0)	72.2 (10.9)	71.3 (10.7)	73.4 (11.3)	0.79
Wears glasses*	188 (37%)	36 (51%)	55 (40%)	35 (29%)	62 (34%)	0.16
Normal hearing*	472 (93%)	65 (93%)	130 (96%)	106 (88%)	171 (95%)	0.23
Hypertension						0.69
Good	43 (8.5%)	7 (26%)	15 (11%)	13 (11%)	8 (4.4%)	
Intermediate	64 (13%)	8 (11%)	16 (12%)	23 (19%)	17 (9.4%)	
Poor	106 (21%)	17 (24%)	25 (18%)	36 (30%)	28 (16%)	
Unknown	293 (58%)	38 (54%)	80 (59%)	48 (40%)	127 (71%)	
Aware of hypertension	134 (27%)	20 (29%)	34 (25%)	42 (35%)	38 (21%)	0.080
Years with hypertension*	9.6 (7.0)	7.0 (8.5)	8.0 (4.2)	9.5 (6.9)	11.5 (7.8)	0.82
In hypertension treatment	82 (16%)	15 (21%)	20 (15%)	21 (18%)	26 (14%)	0.045
Hypertension controlled	81 (16%)	15 (21%)	22 (16%)	27 (23%)	17 (9.4%)	0.93
**Abnormal neurological exam results** *	107 (21%)	15 (21%)	13 (9.6%)	24 (20%)	55 (31%)	0.0004
**Functional evaluation total**	42.7 (10.8)	42.3 (11.6)	45.0 (9.0)	42.8 (12.0)	40.8 (10.7)	0.15
**Cumulative evaluation of disease – mild to severe impairment** *						
Heart	21 (4.2%)	3 (4.3%)	9 (6.6%)	2 (1.7%)	7 (3.9%)	0.11
Vascular	86 (17%)	15 (21%)	18 (13%)	25 (21%)	28 (16%)	0.46
Hematopoietic	4 (0.79%)	0 (0%)	0 (0%)	2 (1.7%)	2 (1.1%)	0.54
Respiratory	15 (3.0%)	0 (0%)	2 (1.5%)	5 (4.2%)	8 (4.4%)	0.089
Eyes, ears, nose, throat	192 (38%)	26 (37%)	45 (33%)	50 (42%)	71 (39%)	0.84
Upper gastrointestinal	61 (12%)	9 (13%)	14 (10%)	18 (15%)	20 (11%)	0.34
Lower gastrointestinal	29 (5.7%)	4 (5.7%)	2 (1.5%)	11 (9.2%)	12 (6.7%)	0.046
Renal	3 (0.59%)	0 (0%)	0 (0%)	2 (1.7%)	1 (0.56%)	0.42
Liver	3 (0.59%)	1 (1.4%)	0 (0%)	1 (0.83%)	1 (0.56%)	0.42
Urogenital	54 (11%)	11 (16%)	9 (6.6%)	18 (15%)	16 (8.9%)	0.11
Musculoskeletal	108 (21%)	18 (26%)	21 (15%)	37 (31%)	32 (18%)	0.022
Endocrine	21 (4.2%)	3 (4.3%)	4 (2.9%)	5 (4.2%)	9 (5.0%)	0.42
Neurological	30 (5.9%)	4 (5.7%)	2 (1.5%)	10 (8.3%)	14 (7.8%)	0.030
Psychiatric	3 (0.59%)	1 (1.4%)	0 (0%)	1 (0.83%)	1 (0.56%)	0.45
**Creatinine, mg/dL**						
Mean (SD)	0.90 (0.30)	0.90 (0.30)	0.90 (0.30)	0.90 (0.40)	0.90 (0.30)	0.75
Median (IQR)	0.85 (0.30)	0.85 (0.35)	0.84 (0.34)	0.87 (0.28)	0.84 (0.28)	0.62
**eGFR Labs**						
MDRD	71.9 (16.2)	74.4 (16.7)	72.0 (15.3)	69.7 (16.7)	72.3 (16.2)	0.30
**Neuropsychiatric**						
**Experience of war** *	220 (44%)	26 (37%)	67 (49%)	55 (46%)	72 (40%)	0.19
**Traumatic event in last 10 years** *	300 (59%)	37 (53%)	79 (58%)	70 (58%)	114 (63%)	0.54
**Smoker** *	60 (12%)	8 (11%)	18 (13%)	15 (13%)	19 (11%)	0.80
**# of years smoking**	1.4 (6.2)	2.0 (6.7)	2.0 (7.0)	1.3 (6.8)	0.8 (4.8)	0.13
**Frequency of alcohol intake** *				0.14		
Never	4 (0.79%)	0 (0%)	1 (0.74%)	0 (0%)	3 (1.7%)	
Once a month	105 (21%)	18 (26%)	31 (23%)	24 (20%)	32 (18%)	
2 to 4 times a month	31 (6.1%)	5 (7.1%)	16 (12%)	3 (2.5%)	7 (3.9%)	
2 to 3 times per week	22 (4.4%)	4 (5.7%)	11 (8.1%)	4 (3.3%)	3 (1.7%)	
4+ times a week	15 (3.0%)	4 (5.7%)	4 (2.9%)	3 (2.5%)	4 (2.2%)	
Unknown	329 (65%)	39 (56%)	73 (54%)	86 (72%)	131 (73%)	
**Sleep disorder** *	155 (31%)	24 (34%)	36 (27%)	40 (33%)	55 (31%)	0.47
**SDOH total**	6.2 (2.7)	7.5 (2.3)	6.4 (2.7)	5.9 (2.8)	5.7 (2.8)^†^	<0.0001
**Resiliency total**	19.7 (3.5)	21.3 (2.4)	21.0 (2.6)	18.0 (4.0)^†^	19.2 (3.6)	<0.0001
**Dimension of poverty total**	6.1 (2.7)	4.7 (2.7)	5.8 (2.6)	6.8 (2.4)^†^	6.5 (2.8)^†^	<0.0001

* Results presented as n (%). .

† Indicates significant pairwise association between the neurological status category and cognitively normal (the reference group).

**TABLE 3:** Cohort cognitive assessments stratified by neurological status.

Variables, mean (SD)	Overall (*n* = 506)	CU (*n* = 70; 14%)	MCI (*n* = 136; 27%)	AD (*n* = 120; 24%)	OD (*n* = 180; 36%)	*p*-value

MCA	19.4 (5.1)	24.2 (3.6)	22.0 (2.9)	15.8 (5.3)^†^	18.2 (4.2)^†^	< 0.0001
CDR	3.3 (3.5)	1.3 (1.5)	2.0 (1.9)	5.5 (4.5)^†^	3.5 (3.4)^†^	<0.0001
**Psychomotricity**						
Sequential movements – dominant hand	7.8 (3.2)	8.7 (2.7)	8.7 (2.3)	6.8 (3.7)	7.5 (3.4)	<0.0001
Sequential movements – non-dominant hand	7.9 (3.5)	8.2 (3.3)	9.1 (2.5)	6.8 (4.0)	7.6 (3.6)	<0.0001
Tandem movement	11.0 (2.8)	11.9 (0.6)	11.6 (2.1)	9.8 (4.1)^†^	11.0 (2.8)	<0.0001
**Attention**						
Mancala – total points	4.1 (2.0)	5.6 (1.4)	4.8 (1.6)	2.9 (2.0)^†^	3.7 (1.9)^†^	<0.0001
Mancala – total correct	27.5 (13.8)	37.7 (10.1)	34.2 (11.1)	18.9 (13.1)^†^	24.2 (12.5)^†^	<0.0001
Mental reversal – total points	4.2 (2.2)	5.9 (1.9)	5.1 (1.8)	2.7 (2.2)^†^	3.9 (1.9)^†^	<0.0001
Mental reversal – total correct	26.0 (15.3)	39.1 (12.5)	32.5 (12.8)^†^	15.0 (13.3)^†^	23.4 (13.2)^†^	<0.0001
**Working memory**						
African market test	14.3 (10.6)	17.9 (20.2)	14.3 (5.3)	12.4 (5.8)	12.9 (6.3)	0.024
**Ideational praxis**	4.4 (0.9)	4.7 (0.5)	4.5 (0.7)	4.1 (1.1)^†^	4.3 (0.9)^†^	<0.0001
**Fluency**						
Phonemic	5.5 (3.9)	8.2 (4.1)	6.0 (3.8)^†^	4.0 (3.5)^†^	5.0 (3.6)^†^	<0.0001
Semantic	10.9 (4.4)	14.0 (3.7)	11.7 (4.1)^†^	8.9 (4.0)^†^	10.3 (4.4)^†^	<0.0001
**Learning and memory**						
ALMT trial 1	4.8 (1.7)	5.9 (1.5)	5.2 (1.4)^†^	3.8 (1.6)^†^	4.8 (1.7)^†^	<0.0001
ALMT trial 2	6.4 (1.8)	7.6 (1.7)	6.9 (1.6)^†^	5.4 (1.8)^†^	6.2 (1.7)^†^	<0.0001
ALMT trial 3	7.2 (2.1)	9.0 (1.4)	7.8 (1.5)^†^	5.9 (2.1)^†^	6.9 (2.0)^†^	<0.0001
ALMT delayed recall	4.9 (2.7)	7.7 (1.7)	5.9 (2.0)^†^	2.3 (2.1)^†^	4.9 (2.2)^†^	<0.0001
ALMT force choice	10.8 (2.1)	11.7 (0.8)	11.4 (1.3)	9.3 (3.1)^†^	10.9 (1.7)	<0.0001
AVMT trial 1	3.6 (3.2)	6.3 (3.7)	4.9 (3.3)	2.0 (2.2)^†^	2.6 (2.2)^†^	<0.0001
AVMT trial 2	4.9 (3.9)	8.4 (3.8)	6.8 (3.8)	2.8 (2.7)^†^	3.6 (3.0)^†^	<0.0001
AVMT trial 3	6.0 (4.6)	10.9 (3.9)	8.2 (4.3)^†^	3.4 (3.1)^†^	4.2 (3.5)^†^	<0.0001
AVMT delayed recall	5.7 (4.5)	11.1 (3.9)	7.7 (4.2)^†^	3.0 (3.2)^†^	4.0 (3.2)^†^	<0.0001
AVMT forced choice	3.9 (0.5)	4.0 (0.0)	3.9 (0.3)	3.7 (0.8)^†^	3.8 (0.6)	<0.0001
ASMT trial 1	6.7 (4.2)	10.1 (3.9)	8.3 (3.8)	3.9 (3.6)^†^	6.0 (3.5)^†^	<0.0001
ASMT trial 2	9.4 (5.1)	14.3 (3.5)	11.6 (4.2)^†^	5.9 (4.3)^†^	8.1 (4.4)^†^	<0.0001
ASMT trial 3	10.6 (5.5)	16. 0 (3.6)	13.2 (4.5)^†^	6.9 (4.7)^†^	9.1 (4.8)^†^	<0.0001
ASMT delayed recall	8.8 (5.4)	14.4 (4.0)	11.1 (4.3)^†^	4.9 (4.3)^†^	7.6 (4.6)^†^	<0.0001
ASMT forced choice	4.0 (1.3)	4.8 (0.4)	4.6 (0.9)	3.3 (1.5)^†^	3.8 (1.2)^†^	<0.0001
**Executive functions**						
Proverb test	2.6 (2.5)	5.3 (2.2)	3.0 (2.3)^†^	1.8 (2.4)^†^	1.7 (2.0)^†^	<0.0001
African card game	12.5 (5.3)	16.4 (4.5)	13.7 (4.5)^†^	10.1 (4.7)^†^	11.7 (5.4)^†^	<0.0001
**Visuospatial perception**	5.7 (2.5)	7.2 (1.7)	6.3 (1.9)	4.8 (3.3)^†^	5.3 (2.1)^†^	<0.0001
**Emotion recognition**	3.5 (1.2)	3.9 (1.1)	3.9 (1.0)	2.9 (1.4)^†^	3.6 (1.2)	<0.0001
**Intelligence**						
Verbal intelligence	21.8 (12.6)	29.8 (12.7)	23.9 (11.6)	18.4 (12.7)^†^	19.4 (11.6)^†^	<0.0001
Non-verbal matrix test	5.6 (3.9)	9.3 (4.6)	6.4 (3.5)^†^	3.9 (3.5)^†^	4.7 (3.2)^†^	<0.0001
Full scall IQ-2	71.3 (15.5)	83.5 (17.6)	73.6 (14.1)^†^	66.8 (14.3)^†^	68.1 (13.8)^†^	<0.0001

† Indicates significant pairwise association between the neurological status category and cognitively normal (the reference group).

## Data Availability

The raw data supporting the conclusions of this article will be made available by the authors, without undue reservation.

## Highlights

- APOE *ε*4 was more frequent in dementia cases than in healthy controls in a DRC cohort.
- APOE *ε*4 carriers showed greater cognitive decline, especially in memory and visuospatial skills.
- Neuroimaging findings revealed increased hippocampal atrophy and cortical thinning in carriers.
- Plasma biomarkers in dementia showed higher APOE *ε*4 ≤ 40, phosphorylated tau 181, neurofilament light chain, and TNF-*α* levels.
- Findings underscore APOE *ε*4’s impact on neurodegeneration in African populations.

## RESEARCH IN CONTEXT

1. **Systematic review**: We reviewed the literature through PubMed and regional sources to identify studies of dementia and cognitive aging in sub-Saharan Africa. Most prior work has been cross-sectional, with limited biomarker integration and virtually no longitudinal studies in French-speaking African countries.
2. **Interpretation**: Our study establishes the first large-scale, community-based dementia cohort in the Democratic Republic of the Congo. By integrating neuropsychological, neurological, social, and biomarker assessments with environmental exposures, we provide novel baseline data on risk factors and phenotypic profiles of AD and related dementias in an underrepresented population. These findings expand the global evidence base and highlight the feasibility of complex dementia research in low-resource contexts.
3. **Future directions**: Ongoing follow-up will allow evaluation of incident dementia, disease progression, and biomarker trajectories. Future research should address gene–environment interactions, resilience factors, and culturally valid diagnostic frameworks to improve early detection, intervention, and global dementia equity.
